# Case Report: Homozygous *KISS1R* mutation associated with congenital hypogonadotropic hypogonadism in two siblings: pulsatile GnRH therapy restores pituitary architecture and induces pubertal development

**DOI:** 10.3389/fmed.2026.1821097

**Published:** 2026-04-30

**Authors:** Rongwan Sun, Xiaotian Lei, Guiliang Peng, Jing Zhu, Liu Chen, Min Long

**Affiliations:** 1 Department of Endocrinology, The First Affiliated Hospital (Southwest Hospital) of Army Medical University, Chongqing, China; 2 Department of Obstetrics and Gynecology, Xinqiao Hospital, Army Medical University, Chongqing, China; 3 Third Military Medical University (Army Medical University), Chongqing, China

**Keywords:** hypogonadotropic hypogonadism, KISS1R mutation, pituitary hypoplasia, pubertal development, pulsatile GnRH therapy

## Abstract

Congenital hypogonadotropic hypogonadism (CHH) is a rare disorder characterized by deficient production, secretion, or action of gonadotropin-releasing hormone (GnRH), the central regulator of the reproductive axis. We report two siblings from a consanguineous family with CHH caused by a homozygous nonsense mutation (c.182C > A; p. Ser 61Ter) in exon 1 of *KISS1R* gene. The 15-years-old male proband presented with absent puberty, micropenis, and gynecomastia. Endocrine evaluation revealed low gonadotropin levels and brain magnetic resonance imaging (MRI) showed pituitary hypoplasia. His 12-years-old sister exhibited puberty delays, with absent breast development and pubic hair. The male patient received 1 year of pulsatile GnRH therapy, resulting in significant clinical improvements, including markedly increased testicular volume (from 0.9 to 5.6 mL), penile growth, and the resolution of gynecomastia. Notably, follow-up imaging demonstrated morphological recovery of the pituitary gland, with an increase in height from 3.5 to 4.5 mm, accompanied by the successful induction of spermatogenesis. This excellent response to GnRH underscores its efficacy in restoring fertility, particularly when initiated in early adolescence, and highlights the value of genetic diagnosis in consanguineous families.

## Introduction

Puberty is a pivotal developmental stage marked by the activation of the hypothalamic-pituitary-gonadal (HPG) axis, which drives sexual maturation and the development of secondary sexual characteristics. This cascade is governed by the pulsatile secretion of gonadotropin-releasing hormone (GnRH) from the hypothalamus, stimulating pituitary release of luteinizing hormone (LH) and follicle-stimulating hormone (FSH) (1).

Congenital hypogonadotropic hypogonadism (CHH) is a rare pubertal disorder resulting from deficient GnRH secretion or action, leading to low sex-steroid levels and low or inappropriately normal gonadotropins. The condition typically manifests during adolescence as failure to initiate or complete spontaneous puberty (2). Etiologically, CHH is highly heterogeneous and can be attributed to pathogenic variants in more than 40 genes involved in GnRH neurons development or migration- often associated with anosmia (Kallmann Syndrome)- or in genes regulating GnRH neuronal function (normosmic CHH) (3), including GNRHR, KISS1R, PROP1 (4, 5). Affected males may present at birth with micropenis or cryptorchidism, while females typically exhibit absent breast development or primary amenorrhea (6). Diagnosis is based on hormonal profiles showing low serum testosterone or estradiol together with low or inadequately normal LH and FSH levels (6). Although pituitary anatomy is frequently normal in CHH, magnetic resonance imaging (MRI) in some patients reveals anterior pituitary hypoplasia (7, 8), the precise correlation between specific genetic variants and these structural findings remains under investigation. Management aims to induce secondary sexual characteristics through sex–steroid replacement or to restore fertility using pulsatile GnRH therapy or gonadotropin injections (2, 6).

KISS1R (also known as GPR54), located on chromosome 19p13.3, encodes a 398–amino–acid G–protein–coupled receptor. Its mRNA is prominently expressed in the hypothalamus, particularly on GnRH–secreting neurons (9). Binding of its ligand, kisspeptin, activates the Gαq/11 signaling pathway, stimulating phospholipase C and intracellular calcium mobilization, which ultimately promotes GnRH release into the portal circulation (10). Inactivating variants in KISS1R disrupt this essential signaling, impair GnRH secretion, and cause profound hypogonadism (11, 12). Genotype–phenotype correlations are complex: although most patients present with complete pubertal failure, some retain partial HPG axis function, resulting in “leaky” phenotypes or variable responses to GnRH therapy. Understanding these relationships is critical for tailoring long-term treatment.

Here, we describe a consanguineous family in which two siblings carry a novel homozygous pathogenic variant in KISS1R (NM_032551.5:c.182C > A, p.Ser61Ter). We detail their clinical presentations, including the uncommon finding of pituitary hypoplasia on MRI, and report the successful recovery of gonadal function–along with morphological pituitary improvement and induced spermatogenesis–in the male proband following 1 year of pulsatile GnRH therapy.

## Case presentation

**FIGURE 1:**
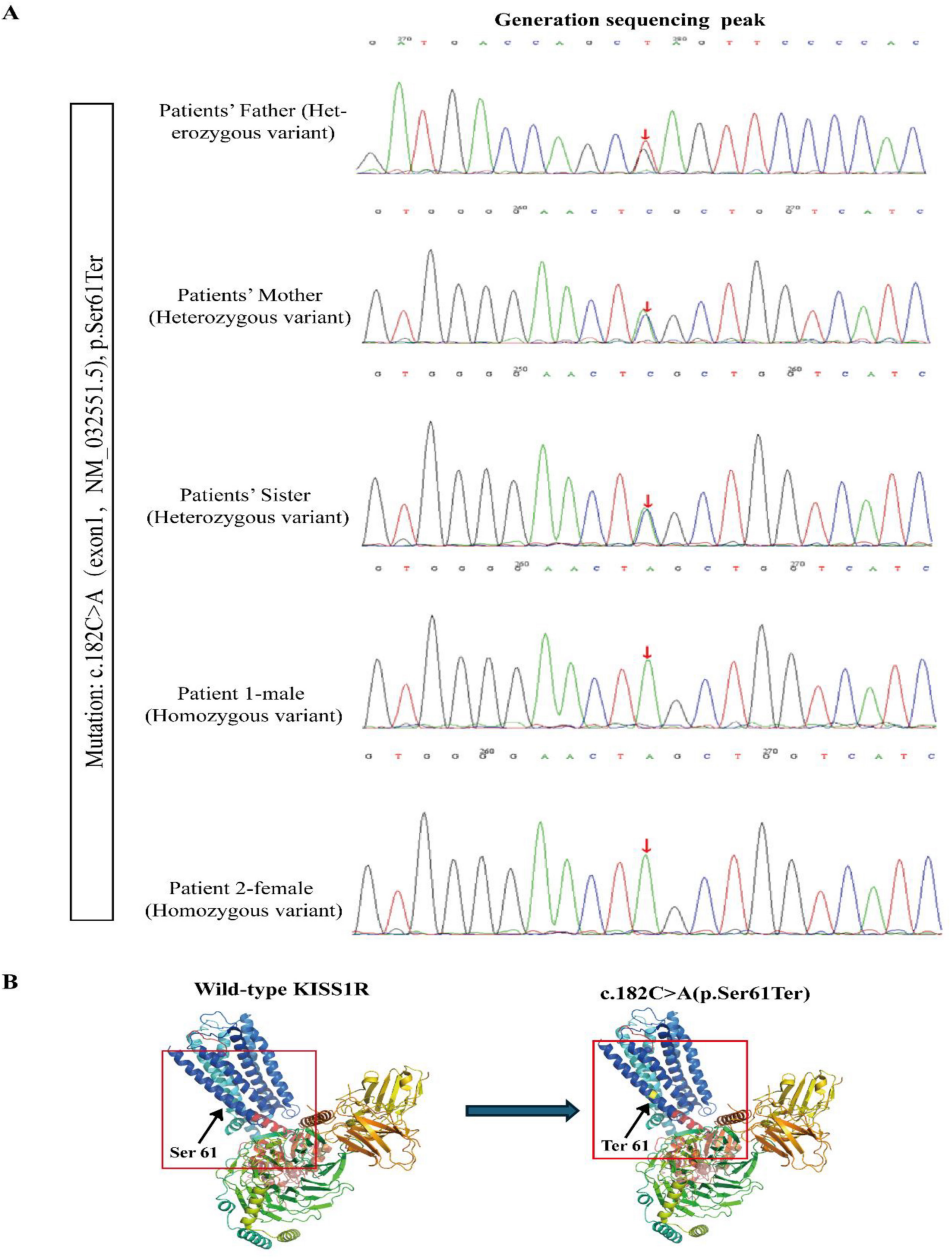
Sanger sequencing and the 3D protein modeling diagram. **(A)** The diagram of Sanger sequencing showed the homozygous mutation (c.182C > A, p. Ser 61 Ter) in *KISS1R* gene in Patients’ family. **(B)** The effect of the p. Ser 61 Ter mutation as shown in a 3D protein modeling. (Right panel): wild-type *KISS1R*, (left panel), p. Ser 61 Ter mutant. p. Ser 61 Ter mutant is marked in yellow of red frame.

Case 1: A 15-years-old male, initially presented with a bone age of 14 years. He was 165 cm tall, weighted 73 kg (BMI 26.8 kg/m^2^), and was at Tanner stage I for pubic hair and genitalia. Physical examination confirmed significant hypogonadism, manifesting as micropenis (1.5 cm), bilaterally small testicular volumes (0.9 ml each), and notable bilateral gynecomastia (glandular tissue thickness: 17 mm on the right, 19 mm on the left). Brain MRI revealed pituitary hypoplasia, with a height of 3.5 mm, olfaction was normal (Table 1). The patient had been receiving testosterone undecanoate therapy at a other hospital since 2008, which was discontinued on the day of admission. Endocrine profile was conducted upon hospital admission, (LH: 0.15 mIU/ml, FSH: 0.25 mIU/ml, Table 1) supporting a hypothalamic defect. A GnRH stimulation test revealed that basal gonadotropin levels were low (LH 0.09 mIU/ml, FSH 0.25 mIU/ml), and a measurable response (peak LH 0.52 mIU/mL, peak FSH 1.03 mIU/mL; Supplementary Figure 1A) was observed, indicating preserved pituitary-gonadal reserve and supporting the suitability of pulsatile GnRH therapy. Conversely, a Human Chorionic Gonadotropin (hCG) stimulation test confirmed impaired Leydig cell function at baseline, with testosterone rising minimally from 0.68 to 0.56 ng/mL (Supplementary Figure 1B). Genetic analysis identified a homozygous pathogenic variant in KISS1R (NM_032551.5:c.182C > A; p.Ser61Ter), classified as likely pathogenic (PVS1 + PM2) by ACMG guidelines (Figure 1A,B).

**TABLE 1:** Clinical features and hormonal parameters of the proband and his sister upon hospital admission.

Subject (showed in Figure 1) Characteristic	Patients V-1 (Proband)	Patients V-3	Normal range for parameters
Gender	Male	Female	/
Age (years)	15	12	/
Bone age (years)	14	9	Matched chronological age
Height (cm)	165	130	/ /
BMI (kg/m^2^)	26.8	17.75	18–24
Pituitary MRI	Pituitary hypoplasia (height 3.5 mm)	NA	/
Ultrasound findings	Breasts-glandular development observed Scrotum-bilateral testes measured smaller than normal (Testicle size: R-0.9 ml, L-0.9 ml)	Uterus-hypoechoic area posterior to the bladder (Underdeveloped uterus?)	/
FG (mmol/L)	/	5.88	3.9–6.1
IGF-1 (ng/ml)	490.40	413.40	132–480
ACTH (pg/ml)	69.00	58.31	5–60
GH (ng/ml)	1.82	1.13	0–5
PRL (ng/ml)	12.33	3.43	3.34–26.72
LH (mIU/ml)	0.15	0.18	M: 1.24–8.62
FSH (mIU/ml)	0.25	1.29	M: 1.27–19.26
T (ng/ml)	1.02	0.0	**/**
E2 (pg/ml)	24.70	12.27	M: 0–38.95
P4 (ng/ml)	1.04	0.26	M: 0.1–0.84
Cortisol rhythm test	Cortisol (nmol/L)	Cortisol (nmol/L)	
8:00	789.51	/	165–441
16:00	277.29	/	55–248
24:00	150.36	/	55–138
TSH (μIU/ml)	2.97	3.91	0.6–4.84
T3 (nmol/L)	2.62	3.47	1.43–3.55
T4 (nmol/L)	81.90	135	77.1–178
FT3 (pmol/L)	6.68	8.16	3.88–8.02
FT4 (pmol/L)	13.90	17.2	12.5–21.5

BMI, body-mass index (BMI is the weight in kilograms divided by square of the height in meters); NA, no abnormal; FG, fasting glucose; IGF-1, insulin-like growth factor-1; ACTH, adrenocorticotropic hormone; GH, growth hormone; PRL, prolactin; LH, luteinizing hormone; FSH, follicle-stimulating hormone; T, testosterone; E2, estradiol; P4, progesterone; F, female; M, male. Assessment of cortisol diurnal rhythm was performed under normal dietary conditions. Blood samples were collected at 08:00, 16:00, and 24:00 (midnight) to evaluate the circadian variation in cortisol secretion. Endocrine profile was performed upon hospital admission. Following an overnight fast, a single venous blood sample was obtained for measurement of sex hormones, thyroid function tests, growth hormone (GH), and insulin-like growth factor-1 (IGF-1).

Case 2: The 12-years-old female sibling, presented with markedly delayed bone age (9 years). Her height was 130 cm and weight was 30 kg (BMI 17.75 kg/m^2^). Physical examination indicated complete absence of pubertal development (Tanner I). Pelvic ultrasound revealed an underdeveloped uterus and pituitary MRI was normal (Table 1). Serum gonadotropin concentrations measured at hospital admission were as follows: LH, 0.18 mIU/mL; and FSH, 1.29 mIU/mL (Table 1). A GnRH stimulation test showed baseline gonadotropin levels were very low (LH 0.23 mIU/mL, FSH 1.68 mIU/mL) while the peak of LH and FSH peaking at 2.61 and 8.3 mIU/mL, respectively (Supplementary Figure 1C). Estradiol was extremely low (7.88 pg/mL), consistent with a prepubertal gonadal state. Following consecutive injections of human menopausal gonadotropin (HMG), estradiol levels began to rise from day 2, reaching 33.05 pg/mL by day 3 (Supplementary Figure 1D), indicating preserved ovarian follicular reserve and responsiveness to gonadotropin stimulation. Genetic analysis confirmed the same homozygous *KISS1R* variant (NM_032551.5:c.182C > A; p.Ser61Ter) in this patient.

## Genetic findings and clinical feature of patients’ family

**FIGURE 2:**
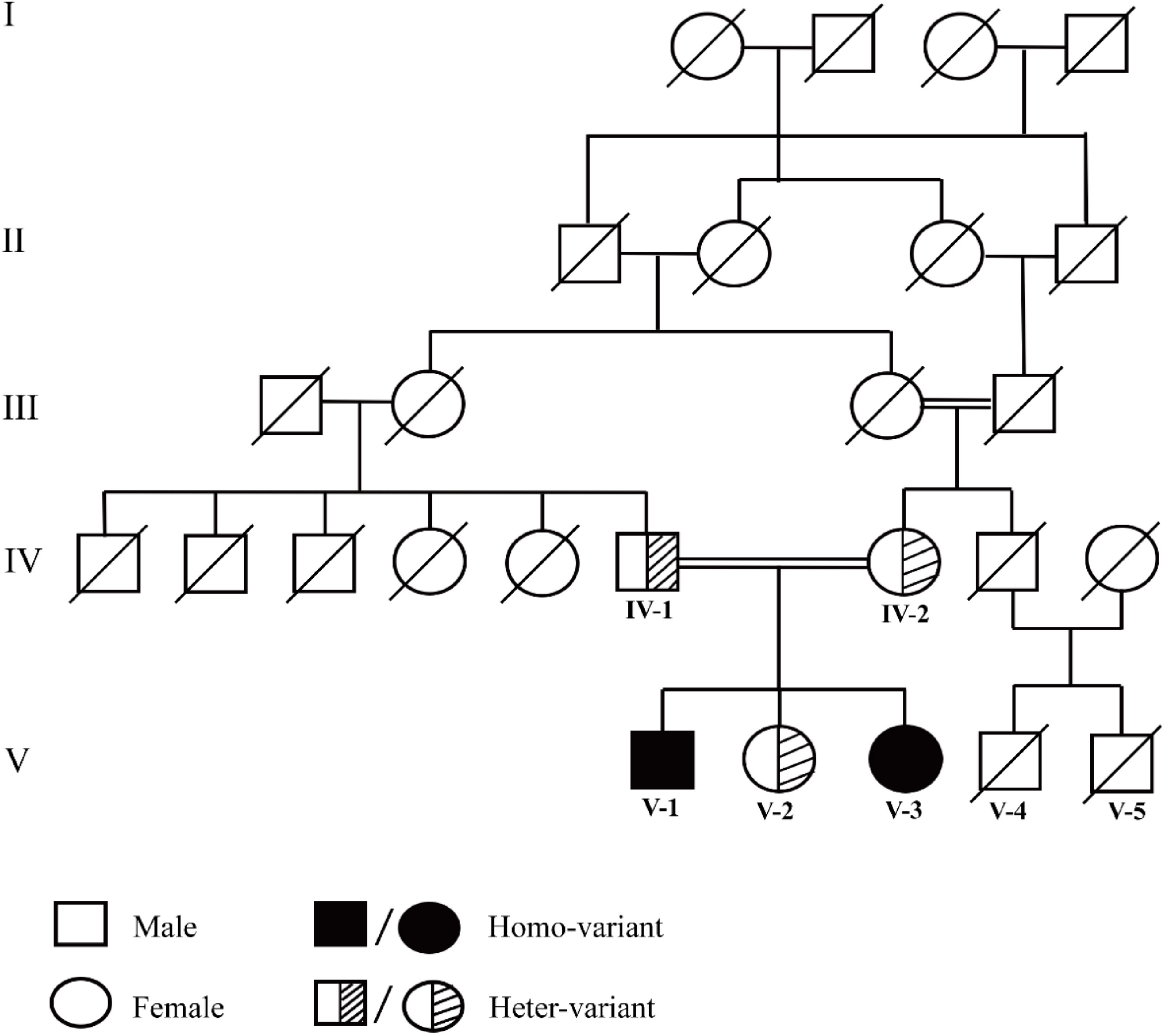
Pedigree analysis of the patients’ family. Black solid indicates affected family members (homozygous variant). Stripes one is a heterozygotes variant. Slash means no genetic testing was done.

Whole-exome sequencing of the proband (V-1) identified a novel homozygous nonsense variant in exon 1 of the *KISS1R* gene (c.182C > A), resulting in replacement of Serine by terminator (p.Ser61Ter). Sanger sequencing confirmed the same homozygous variant in his younger sister (V3). The proband’s father (IV-1), mother (IV-2), and older sister (V-2) were heterozygous carriers of this variant (Figures 1A,B, 2). The family pedigree and genetic results demonstrate that the homozygous variant segregates with the CHH phenotype in this consanguineous family, strongly supporting its pathogenicity.

Upon detailed pedigree analysis, the proband’s father (individual IV-1) exhibited normal growth and development with no evidence of gonadal dysfunction. The mother (individual IV-2) reported a history of delayed pubertal onset, characterized by delayed thelarche and pubarche, with menarche occurring at age 16 years; her adult stature is 150 cm. The proband’s older sister (individual V-2) is 18 years old with a height of 148 cm and weight of 62 kg. She attained menarche at age 11 years and had reportedly normal sex hormone levels on previous evaluation. She has a history of insulin resistance, for which she was previously treated with metformin (now discontinued).

## Treatment

Case 1: The patient was treated with pulsatile GnRH (gonadorelin 10 μg every 90 min subcutaneous injected via a portable pump) without concurrent sex steroid supplementation.

Case 2: Given her prepubertal status and preserved GnRH responsiveness, active pharmacologic treatment was not initiated. She has been monitored clinically with regular auxological and hormonal assessments.

## Outcome and follow-up

Case 1

**FIGURE 3:**
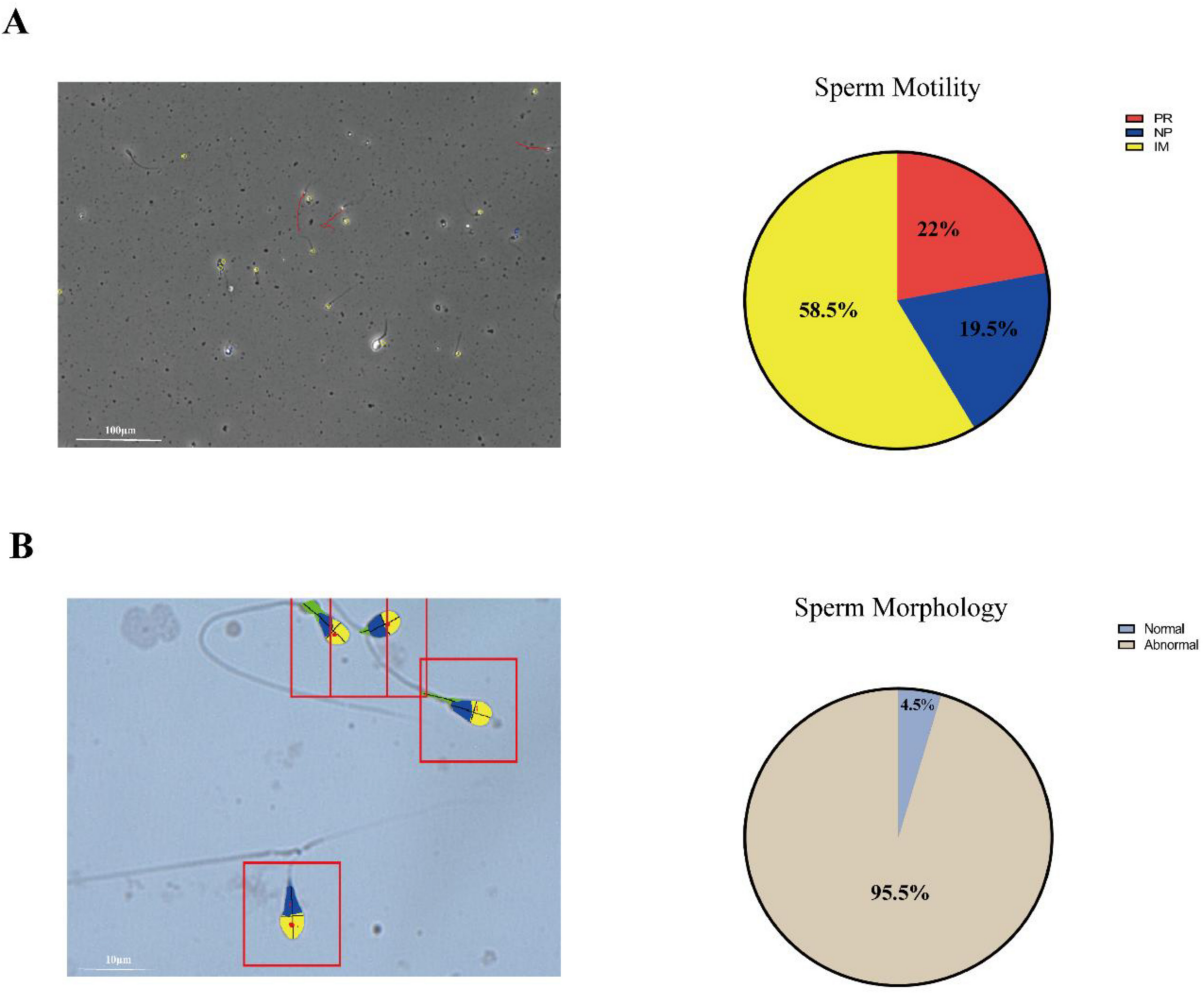
The sperm motility and morphology of Case 1 with 1-year GnRH therapy had both reached normal reference ranges. **(A)** Sperm motility and **(B)** morphology assessment. PR, prograssive; NP, non-prograssive; IM, inactive motile.

The patient demonstrated excellent treatment adherence, receiving uninterrupted pulsatile GnRH pump therapy throughout the follow-up duration without any episodes of discontinuation or missed doses. He showed marked clinical improvement: testicular volume increased from 0.9 to 5.6 ml, penile length increased from 1.5 to 4.5 cm, and Tanner stage advanced from I to II. Gynecomastia resolved completely. Hormones parameters improved significantly LH increased from 0.15 to 8.09 mIU/ml, FSH from 0.25 to 3.85 mIU/ml, and testosterone from 1.02 to 2.08 ng/ml. Follow-up MRI revealed morphological recovery of the pituitary gland, with its height increasing from 3.5 to 4.5 mm. This structural improvement was accompanied by functional restoration of spermatogenesis, as confirmed by semen analysis: progressive motility was 41.5% (reference: PR + NP > 40%, Figure 3A) and normal morphology > 4.5% (reference: >4.0%, Figure 3B).

Case 2

Over 1 year of observation, her height increased by 5 to 135 cm, but no spontaneous pubertal development (Tanner stage I) occurred. She continues to be monitored for spontaneous pubertal onset.

## Discussion

We identified a novel homozygous *KISS1R* variant, c.182C > A (p.Ser61Ter), in two affected siblings from a consanguineous family, establishing its causative role in their congenital hypogonadotropic hypogonadism (CHH). Inactivating variants in *KISS1R* are a known cause of normosmic CHH, first described in 2003 (13, 14), and are estimated to underlie approximately 5% of cases, particularly in consanguineous populations (15). The p.Ser61Ter variant introduces a premature termination codon, predicted to result in a severely truncated, non-functional receptor. Such loss-of-function mechanisms typically lead to complete kisspeptin signaling failure, aligning with the profound hypogonadism observed in the male proband (16). Pedigree analysis is consistent with an autosomal recessive inheritance pattern, which is characteristic of severe, inactivating *KISS1R* variants (5). This model is genetically confirmed by Sanger sequencing, which showed that the asymptomatic parents and an unaffected sister are heterozygous carriers, whereas only the two clinically affected siblings are homozygous for the variant.

**FIGURE 4:**
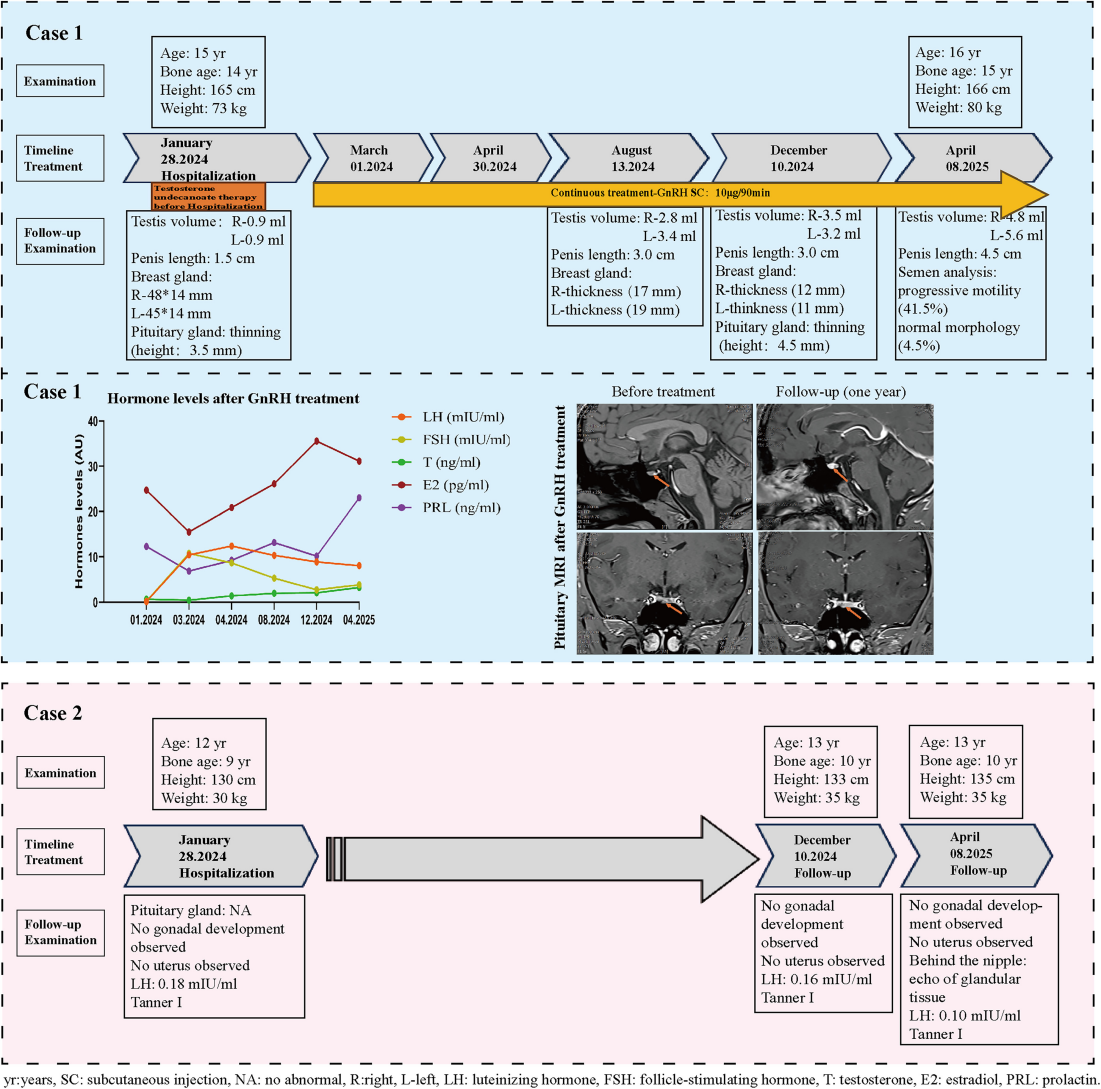
Summarize the changes in follow-up parameters from admission to 1 year GnRH therapy. yr, years; SC, subcutaneous injection; NA, no abnormal; R, right; L, left; LH, luteinizing hormone; FSH, follicle-stimulating hormone; T, testosterone; E2, estradiol; PRL, prolactin.

A notable finding in our proband was pituitary hypoplasia on MRI. The imaging revealed a flattened, lean profile along the posterior and inferior margin of the gland–a morphology often described in empty sella syndrome (17), which can occur as an incidental finding even in individuals with normal pubertal development and androgenization, though it is occasionally associated with hypothalamic-pituitary dysfunction (18). Pituitary MRI is typically normal in patients with *KISS1R* mutations (19). Pituitary hypoplasia is not a classically recognized feature of *KISS1R* mutations, where the defect is presumed to be confined to hypothalamic GnRH activation (20). Notably, the patient had received testosterone supplement therapy undecanoate at other hospital prior to evaluation. We consider that testosterone therapy may potentially accelerate this morphological change through a negative feedback suppression. Our hypothesis is based on the dual-site negative feedback of exogenous testosterone on the HPG axis. First, testosterone suppresses residual GnRH pulsatility via androgen receptor-mediated inhibition of KNDy neurons in the hypothalamus (21). Second, it directly reduces gonadotroph sensitivity to GnRH at the pituitary level (22). In patients with KISS1R mutations, baseline GnRH secretion is severely compromised; exogenous testosterone therefore creates a state of “complete GnRH silence,” accelerating gonadotroph atrophy through apoptosis pathways (e.g., prohibitin-mediated) (23). This explains the “empty sella-like” morphology observed on MRI. Importantly, the morphological recovery following pulsatile GnRH therapy supports that this change is reversible and functional, rather than a developmental structural defect (24). Our observation only represents a phenotypic association within these specific siblings, not definitive proof of causality. Reassuringly, after 1 year of pulsatile GnRH therapy, follow-up MRI demonstrated clear morphological improvement of the pituitary gland (Figure 4).

The phenotypic discordance between the siblings-with the prepubertal sister exhibiting growth delay but a structurally normal pituitary-highlights the variability that can arise even with identical genotypes. Reported cases of biallelic *KISS1R* loss-of-function typically describe normosmic CHH with normal stature in both sexes. Short stature is a consistent feature. Whether the phenotypic heterogeneity we observed reflects true sexual dimorphism remains uncertain and warrants further investigation in model systems.

This report concerns a 15-years-old male proband who presented with a complete absence of pubertal development. Genetic sequencing identified a homozygous pathogenic variant in the *KISS1R* gene, and both parents were confirmed as heterozygous carriers, consistent with an autosomal recessive inheritance pattern. This mutation results in a severe defect in hypothalamic GnRH pulse secretion. Based on this definitive diagnosis of “hypothalamic-type” CHH, we forwent the conventional initial approach of testosterone replacement and instead directly initiated the pathophysiologically targeted intervention–exogenous pulsatile GnRH pump therapy. This decision was aimed not only at inducing secondary sexual characteristics but also at reconstituting the entire hypothalamic-pituitary-gonadal (HPG) axis from its origin, thereby maximizing long-term reproductive potential.

After 1 year of pulsatile GnRH treatment, a comprehensive and coordinated recovery of the patient’s HPG axis was observed, which can be systematically evaluated at four distinct levels: (1) Morphological: Cranial MRI demonstrated recovery of pituitary height from 3.5 to 4.5 mm, directly reflecting reactivation and expansion of the gonadotroph cell population. (2) Endocrine: Gonadotropin (LH: 8.09 IU/L) and testosterone (3.24 ng/mL) levels rapidly normalized to the adult range. (3) Gonadal: Testicular volume increased more than fivefold, from 0.9 to 5.6 ml. (4) Functional: Semen analysis confirmed the presence of spermatozoa with good motility (progressive motility: 41.2%) and a normal morphology rate (4.5%). These results aligns with previous studies showing that pulsatile GnRH can effectively bypass the hypothalamic defect in patients with *KISS1R* mutations, enabling pubertal induction and fertility restoration (25). Thus, the treatment served both as a diagnostic tool confirming hypothalamic origin of the defect and as an effective long-term therapeutic strategy.

## Conclusion

We report phenotypic heterogeneity between siblings carrying the identical homozygous *KISS1R* variant, thereby expanding the recognized clinical spectrum of this rare disorder. Our findings highlight the essential role of genetic diagnosis in consanguineous families with CHH, as it enables precise, pathophysiology–guided management. In the affected male adolescent, pulsatile GnRH therapy comprehensively restored gonadal function, successfully induced spermatogenesis, and promoted morphological recovery of the pituitary gland. This case provides a multidimensional evaluation of early–adolescent pulsatile GnRH therapy, offering valuable insights to inform clinical diagnosis and management.

## Data Availability

The original contributions presented in the study are included in this article/Supplementary material, further inquiries can be directed to the corresponding authors.
