# Supplementary material for: Case Report: Homozygous KISS1R mutation associated with congenital hypogonadotropic hypogonadism in two siblings: pulsatile GnRH therapy restores pituitary architecture and induces pubertal development
Source: Front Med (Lausanne). 2026 Apr 30;13:1821097. doi: 10.3389/fmed.2026.1821097 (PMC13171369; doi:10.3389/fmed.2026.1821097)
Supplement: Supplementary file 1 [file Data_Sheet_1.pdf]

Homozygous *KISS1R* Mutation Associated with Congenital Hypogonadotropic Hypogonadism in Two Siblings: Pulsatile GnRH Therapy Restores Pituitary Architecture and Induces Pubertal Development

Rongwan Sun[1#], Xiaotian Lei[1,2#], Guiliang Peng[1], Jing Zhu[3], Liu Chen[2*], Min Long[1,2*]

Supplementary Figure and Table

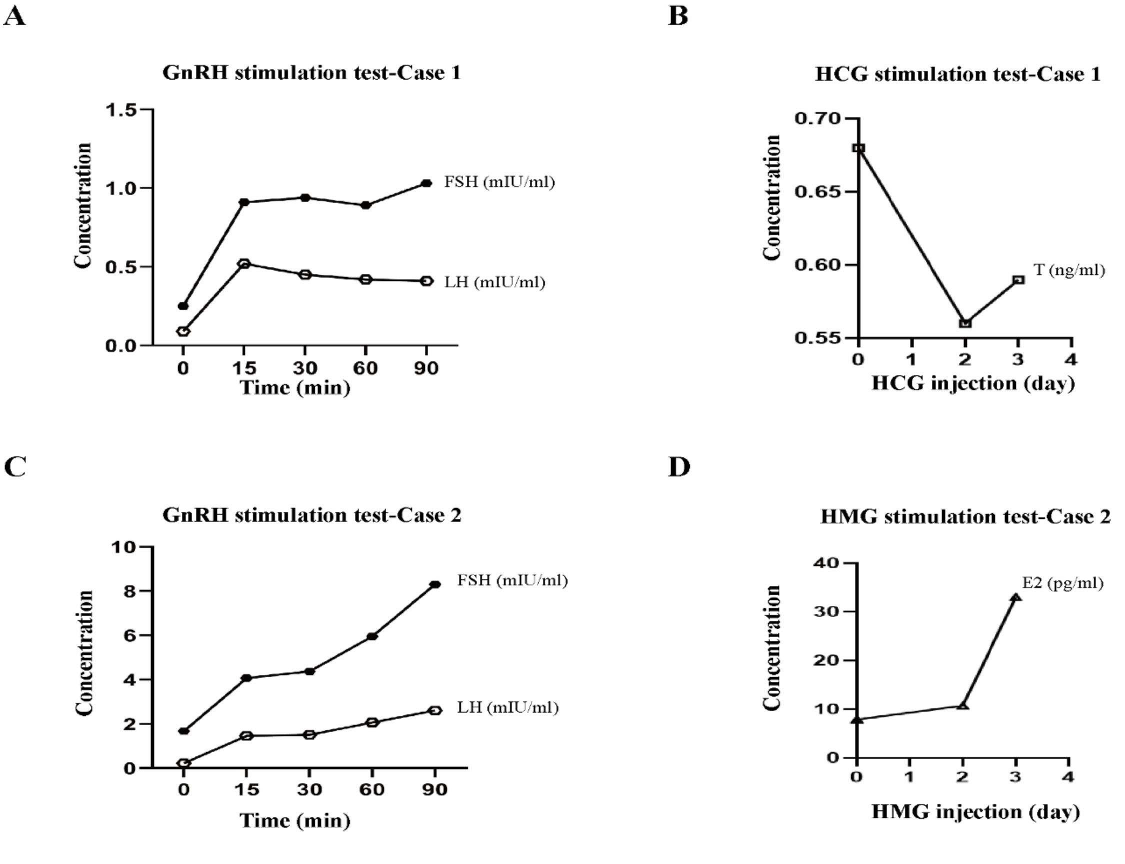

Supplementary Figure 1. Pituitary and Gonadal Stimulation Tests. (A) GnRH stimulation test in the male proband (Case 1) showing a blunted gonadotropin response, with minimal increases in LH and FSH. (B) HCG stimulation test in Case 1, demonstrating a poor testosterone (T) response. (C) GnRH stimulation test in the female sibling (Case 2), showing a normal prepubertal gonadotropin response. (D) HMG stimulation test in Case 2, confirming a normal estradiol (E2) response. **GnRH stimulation test** was performed using triptorelin acetate (GnRH agonist). Baseline blood samples for follicle-stimulating hormone (FSH) and luteinizing hormone (LH) were obtained prior to drug administration. Subcutaneous injection

of triptorelin acetate (0.1 mg) was then administered, and additional blood samples were drawn at 15, 30, 60, and 90 minutes post-injection to assess the dynamic gonadotropin response. **Human chorionic gonadotropin (hCG) stimulation test** was conducted as follows: Baseline serum testosterone and estradiol levels were measured on Day 1 prior to hCG administration. Intramuscular injection of hCG (2000 IU) was subsequently given, and hormone levels (testosterone and estradiol) were remeasured on Days 2 and 3 (24 and 48 hours post-injection).
